# Risk prediction models for diabetic foot ulcer development or amputation: a review of reviews

**DOI:** 10.1186/s13047-023-00610-6

**Published:** 2023-03-16

**Authors:** Anjum S. Kaka, Adrienne Landsteiner, Kristine E. Ensrud, Brittany Logan, Catherine Sowerby, Kristen Ullman, Patrick Yoon, Timothy J. Wilt, Shahnaz Sultan

**Affiliations:** 1 Section of Infectious Diseases, Minneapolis VA Affairs Health Care System, 1 Veterans Drive, Minneapolis, MN 111F55417 USA; 2 Department of Medicine, University of Minnesota Medical School, Minneapolis, MN USA; 3 Evidence Synthesis Program, Minneapolis Veterans Affairs Health Care System, Minneapolis, MN USA; 4 Center for Care Delivery and Outcomes Research, Minneapolis Veterans Affairs Health Care System, Minneapolis, MN USA; 5 Section of General Internal Medicine, Minneapolis VA Affairs Health Care System, Minneapolis, MN USA; 6 Division of Epidemiology and Community Health, School of Public Health, University of Minnesota, Minneapolis, MN USA; 7 Section of Podiatry, Minneapolis VA Affairs Health Care System, Minneapolis, MN USA; 8 Section of Orthopedics, Minneapolis VA Affairs Health Care System, Minneapolis, MN USA; 9 Department of Orthopedic Surgery, University of Minnesota, Minneapolis, MN USA; 10 Division of Gastroenterology, University of Minnesota Medical School, Minneapolis, MN USA

**Keywords:** Risk prediction, Prognostic model, Diabetic foot ulcer, Amputation

## Abstract

**Background:**

In adults with diabetes, diabetic foot ulcer (DFU) and amputation are common and associated with significant morbidity and mortality.

**Purpose:**

Identify tools predicting risk of DFU or amputation that are prognostically accurate and clinically feasible.

**Methods:**

We searched for systematic reviews (SRs) of tools predicting DFU or amputation published in multiple databases from initiation to January, 2023. We assessed risk of bias (ROB) and provided a narrative review of reviews describing performance characteristics (calibration and discrimination) of prognostically accurate tools. For such tools, we additionally reviewed original studies to ascertain clinical applicability and usability (variables included, score calculation, and risk categorization).

**Results:**

We identified 3 eligible SRs predicting DFU or amputation risk. Two recent SRs (2020 and 2021) were rated as moderate and low ROB respectively. Four risk prediction models – Boyko, Martins-Mendes (simplified), Martins-Mendes (original), and PODUS 2020 had good prognostic accuracy for predicting DFU or amputation over time horizons ranging from 1- to 5-years. PODUS 2020 predicts absolute average risk (e.g., 6% risk of DFU at 2 years) and consists of 3-binary variables with a simple, summative scoring (0–4) making it feasible for clinic use. The other 3 models categorize risk subjectively (e.g., high-risk for DFU at 3 years), include 2–7 variables, and require a calculation device. No data exist to inform rescreening intervals. Furthermore, the effectiveness of targeted interventions in decreasing incidence of DFU or amputation in response to prediction scores is unknown.

**Conclusions:**

In this review of reviews, we identified 4 prognostically accurate models that predict DFU or amputation in persons with diabetes. The PODUS 2020 model, predicting absolute average DFU risk at 2 years, has the most favorable prognostic accuracy and is clinically feasible. Rescreening intervals and effectiveness of intervention based on prediction score are uncertain.

## Introduction

In 2019, an estimated 37 million individuals, or 11.3% of the United States (U.S.) population, has diabetes [1]. Many persons with diabetes have additional complications, including neuropathy and peripheral arterial disease, which increase the risk of developing foot ulcers [2, 3]. Between 15–25% of persons with diabetes develop a diabetic foot ulcer (DFU) during their lifetime [4]. Furthermore, a DFU is the greatest risk factor for lower extremity amputation; persons with type 2 diabetes (compared to those without) have a ten-fold higher rate of amputation [5]. The development of a DFU or amputation results in a significant decrease in patients’ quality of life and productivity due to a reduction in physical, social and employment activities [6]. Treatment costs range between $18,600 to $35,100 per DFU, with the U.S. spending $60 billion annually on lower extremity-related care for patients with diabetes [7, 8]. Hence, DFU development or amputation is associated with significant morbidity, mortality, decreased quality of life and productivity, and substantial health care costs.

Since the 1990s, a large body of research has focused on developing tools that predict risk of DFU or amputation with the goal of identifying persons at high-risk who may benefit from early prevention and intervention [8]. Multiple recent systematic reviews (SRs) have described these prediction tools and their performance characteristics [9–11]. This review of reviews was conducted (as part of a larger review) by the Department of Veterans Affairs (VA) Evidence Synthesis Program at the request of the VA National Clinical Orthotic and Prosthetic Program Office. The goal was to identify tools that predict DFU development or amputation and summarize their performance (prognostic accuracy) to help inform clinical practice and policy in the VA Health Care System. Hence, we also evaluated the usability of the tool in busy outpatient clinics. Given the high prevalence of DFUs and amputations in the U.S. and worldwide, risk stratification and prevention would be of great benefit to individuals, health care systems, and society.

## Methods

### Overview

This review of reviews focused on the performance characteristics (e.g., accuracy, external validation, and clinical applicability) of tools that predict development of a new DFU (first or subsequent). It also provides a detailed description of the clinical applicability and usability of top-performing prediction tools based on additional data abstraction from the original studies describing tool development and validation. This review was registered in PROSPERO (http://www.crd.york.ac.uk/PROSPERO/; #CRD42021287645).

### Data sources and search strategy

We searched for peer-reviewed English language SRs from inception to January 13, 2023 in MEDLINE, Embase, and the Cochrane Database of Systematic Reviews. A search filter was applied to limit results to reviews and a language restriction (English) was also used. To supplement the database search, we reviewed reference lists of relevant SRs and sought input for additional studies from our clinical experts or Technical Expert Panel members. Appendix Table 1 shows the detailed search strategy.

### Study selection of eligible reviews

At least two reviewers (A.L., A.K., and C.S.) independently screened titles and abstracts of all identified studies using Distiller SR (Evidence Partners, Ottawa, Canada). Articles included by any reviewer were moved to full-text review. At full-text review, at least two individuals decided independently on inclusion/exclusion; disagreements or inconsistencies were resolved by discussion and input from a third reviewer. Pre-specified eligibility criteria included SRs that evaluated tools predicting DFU (primary or subsequent) or amputation in adults (≥ 18 years of age) with diabetes. Studies were excluded if they were not peer-reviewed full-text SRs or included non-eligible populations such as children.

### Data extraction and quality assessment

Data from all eligible SRs was abstracted by one reviewer (A.L.) and confirmed by a second reviewer (C.S.) using a standardized data extraction form developed with input from review team members based on a priori inclusion, exclusion criteria, as well as populations, interventions and outcomes of interest. This data extraction form was pilot tested before abstraction. We abstracted data on SR title and authors, funding sources, SR characteristics, search dates and strategy, population characteristics, outcome, number of studies and models predicting outcomes of DFU development or amputation, SR limitations, and SR authors’ conclusions on top-performing tools. When information from the review was missing, we accessed the primary publication. Since the included SRs were narrative, we did not identify discrepant or overlapping data. Extracted data was cross-checked by a third reviewer (A.K.) for quality assurance but no formal kappa for agreement was calculated because of the limited number of studies for which data extraction was performed. Risk of bias (ROB) was assessed using the Risk of Bias in Systematic Review (ROBIS) tool [12] and is provided in Appendix Table 2. ROB was assessed by one reviewer, confirmed by a second, and disagreements were resolved by discussions or a third reviewer.

### Data synthesis and analysis

Data was synthesized by one reviewer and confirmed by the second using an electronic form. There were no disagreements. Given the heterogeneity in populations, the varying inclusion/exclusion criteria across reviews and differences in SR methodology, we provided a qualitative or narrative summary of the best performing prediction tools from the included SRs. There is currently no GRADE guidance on assessment of certainty for risk prediction models. The GRADE prognosis project group is currently developing definitive guidance for application to clinical prediction models [13]. We primarily relied on the review authors’ assessments and reporting of prediction tool performance. For tools with good prognostic accuracy, we retrieved and reviewed the primary studies that reported on the original tool development. We categorized tools based on inclusion of a specified prediction horizon, as (i) risk classification systems if they predicted level of risk without a specified time horizon, or (ii) risk prediction models if they predicted risk over a specified time horizon. For risk prediction models, prognostication could be subjective (e.g., high risk at 2 years), relative (e.g., twofold increased risk at 2 years), or absolute (e.g., 6% rate of DFU development at 2 years). Since prediction over a specified time horizon is important for clinical decision-making, including interventions and referral to specialists, we only included risk prediction models for further consideration.

For the top performing risk prediction models, we abstracted prognostic accuracy measures (calibration and discrimination) reported by original studies of tool development (internal/external validation) and the Beulens et al. SR (external validation) [9]. An independent search for all external validation studies for tools was not conducted. Calibration was evaluated using calibration slope and the observed/predicted ratios. Discrimination was evaluated using a C statistic or area under the curve for the receiver operating curve (AUC-ROC) [14]. A C statistic value of 0.5–0.6 was rated as poor, 0.6–0.7 as fair, 0.7–0.8 as good, and ≥ 0.8 as excellent discrimination. A formal risk of bias assessment or evaluation of the primary studies was not performed.

Finally, to determine clinical relevance and usability, we analyzed the number of variables included in each model, ease of obtaining these variables in the busy clinical practice setting, feasibility of score calculation, and categorization of risk.

## Results

**Fig. 1:**
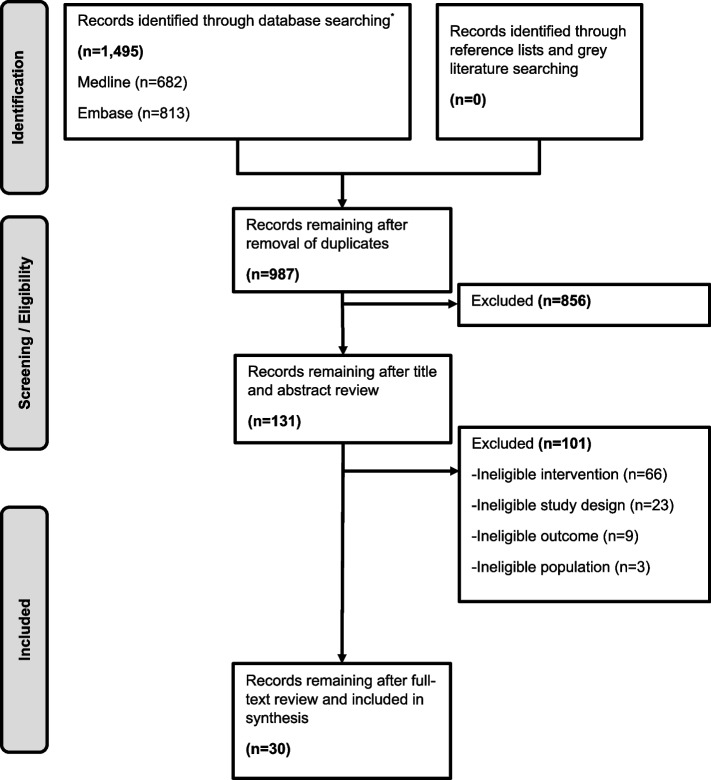
Literature Flow Diagram. ^*^Search through January 13, 2023

Of 1,495 unique citations, 131 articles underwent full-text review of which 30 reviews were deemed eligible for the larger report (Fig. 1) [9–11]. Of these, 3 SRs were eligible for this review of reviews (tools that predict risk of DFU or amputation). Two SRs were rated low ROB [9, 11] and one was rated moderate ROB [10]. Detailed characteristics and conclusions of the 3 SRs are provided in Table 1. Across these three SRs, there was heterogeneity in the populations, models, and outcomes of the included studies, and the SRs reached different conclusions. We first provide results from eligible SRs, then describe performance characteristics of the top performing prediction models (distinguishing models used for risk classification versus risk prediction) and finally we reviewed usability characteristics.

**Table 1:** Overview of systematic reviews evaluating, for patients with diabetes, risk prediction tools for diabetic foot ulcer development or amputation

Author (year); Risk of Bias (ROBIS); Search Dates; Sources; Study type	Population	Outcome	#Studies/ #Models	Limitations	Authors’ Conclusions	Our Conclusions
Beulens et al. (2021) [9]; **Low ROB**^a^; Inception-10/21/2020; PubMed and EMBASE; Systematic review and external validation study; Funding: Dutch Diabetes Research Foundation	Patients with type 2 diabetes	Foot ulcer development, amputation, or neuropathy, or a combination of these over a minimal 1 year follow-up	21/34	(i) Low [5-year] incidence of amputation in the external validation cohort (70/7624; 0.9%), (ii) inability to differentiate between major and minor imputations (missing data), (iii) limited generalizability to populations/settings different from validation cohort (iv) inability to validate models including variables not available in validation cohort	The models by Boyko et al., [14] PODUS 2015, [15] and Martins-Mendes et al., [16] performed well to predict outcomes of either amputation or foot ulcer	PODUS 2015 was developed as a risk classification system with no time horizon for risk prediction. Hence, it was excluded from further consideration. The models by Boyko et al. and Martins-Mendes et al. are prognostic models
Fernandez-Torres et al. (2020) [10]; **Moderate ROB**; Inception- 12/30/2019; PubMed, Scopus, SciELO, CINAHL, Cochrane, PEDro, and EMBASE; Systematic review; Funding: None	Patients with diabetic foot disease including neuropathy, regardless of the type of diabetes	Neuropathy risk, ulceration risk, and diabetic foot ulcer outcome (amputation risk, healing, infection assessment, and measurement)	29/39	Exclusion of tools not published in English, French, Spanish, Portuguese, and Italian, or reporting psychometric characteristics not captured by the review’s inclusion criteria	The Queensland High Risk Foot Form (QHRFF) was valid and reliable for the assessment of ulceration risk	QHRFF was developed as a risk classification system with no time horizon for prediction. Hence, it was excluded from further consideration
Monteiro-Soares et al. (2011) [11]; **Low ROB**; Inception until 4/15/2010; MEDLINE; Systematic review; Funding: None	Patients with diabetes, type unspecified	Foot ulcer development	13/5	Quality assessment, data analysis, and extraction were performed by one reviewer who was not blinded to authors or institutions	The best method for assessment of risk stratification is not immediately apparent	Identical to the authors conclusions

^a^Risk of Bias

### Systematic reviews of prediction tools

The SR by Beulens et al. (low ROB) identified tools that predicted DFU or amputation risk in patients with type 2 diabetes (without a DFU at baseline) with ≥ 1-year follow-up [9]. Beulens et al. identified 21 studies of 34 risk prediction models predicting neuropathy, DFU, or amputation. The commonly used prediction horizons were 1 year and 10 years. The authors also conducted an external validation study of 13 models predicting DFU or amputation using a Dutch cohort of community-dwelling adults with type 2 diabetes mellitus (mean age 67 years, 53% male, 4.1% with a history of DFU or amputation) seen in a primary care clinic (*n* = 7,624) using a 5-year follow-up period. In this external validation cohort, 485 (6.4%) developed a new DFU and 70 (0.9%) underwent amputation during the 5-year follow-up. Among individuals with no history of DFU or amputation (*n* = 7309; 95.9% of entire cohort), 265 (3.6%) developed a DFU and 28 (0.4%) underwent amputation over 5 years. In contrast, among individuals with a prior DFU or amputation (*n* = 315), 220 (69.8%) developed a DFU and 42 (13.3%) underwent amputation over 5 years.

Based on the external validation results, the authors identified top-performing models for:

(i) Predicting new DFU at 5 years: The Boyko [15], PODUS 2015 [16], and Martins-Mendes (original and simplified) [17] models performed well with good to excellent discrimination. Calibration plots for the Martins-Mendes models (original and simplified) demonstrated good agreement between observed and predicted rates in the lower quintiles of predicted risk, but observed risks exceeded predicted risks in the higher quintiles. No calibration plots were presented for the models by Boyko or PODUS 2015.
(ii) Predicting amputation at 5 years: The Martins-Mendes models (original and simplified) performed well with good to excellent discrimination (C statistic 0.81 and 0.78, respectively). Calibration plots for these models for amputation showed results similar to their performance for DFU prediction, i.e., good agreement for amputation prediction between observed and predicted risks in the lower quintiles of predicted risk, but observed risks exceeded predicted risks in the higher quintiles of predicted risk.

The authors concluded that using a combined endpoint of DFU or amputation prediction, the models by Boyko, PODUS 2015, and Martins-Mendes showed good performance and may be applicable for use in clinical practice.

The SR by Fernandez-Torres et al. [10] (moderate ROB) identified clinician-assessment tools for measuring diabetic foot disease related variables which included neuropathy and ulceration risk, and DFU-related variables which included amputation risk, healing, infection assessment, and measurement, applicable to patients with diabetes (type 1 or 2). Studies were excluded if tools did not include psychometric properties in their development or did not provide any measurement properties that met the consensus-based standards for the selection of health measurement instruments (COSMIN) criteria. This SR identified 29 studies of 39 clinician-assessment tools validated for the assessment of diabetic foot disease and DFU-related variables. Prediction horizons were not reported. Thus, measures of calibration and discrimination or absolute risks of diabetic foot disease or DFU related outcomes over a specified time horizon were not reported. ROB of included studies was not reported. Of the 10 scales assessing ulceration risk, the authors identified the Queensland High Risk Foot Form scale (QHRFF) as a valid and reliable instrument for assessing risk of developing a DFU. However, the authors also stated that the psychometric characteristics of QHRFF did not have sufficient strength, because the QHRFF validation study was conducted in only 22 subjects.

The SR by Monteiro-Soares et al. [11] (low ROB) identified risk stratification systems for predicting DFU and identified 13 studies evaluating 5 models. The authors stated that the quality of evidence for these systems was low, as little validation of their predictive ability had been performed. Hence, the authors concluded that the best method for assessment of risk stratification was not immediately apparent.

### Re-classification and performance characteristics of prediction tools

Based on the results and conclusions of the 3 SRs described above, we identified 5 recommended tools to predict DFU or amputation risk: Boyko et al., Martins-Mendes et al. (simplified and original), PODUS 2015, and QHRFF [9–11]. We additionally identified an updated model for PODUS 2015 – PODUS 2020 [8] from a review of the reference lists of SRs. Hence, in total we prioritized 6 tools for further review. For these, we reviewed original studies outlining tool development and ultimately categorized tools as risk classification systems or risk prediction models [8, 15–20] described in Appendix Tables 3 and 4. We determined that PODUS 2015 and QHRFF are best categorized as risk classification systems as they do not specify the time horizon for prediction, hence we excluded these from further consideration. We describe below prognostic accuracy of the following risk prediction models: Boyko, Martins-Mendes (original and simplified), and PODUS 2020. All 4 risk prediction models predict DFU; the 2 Martins-Mendes models also predict amputation. The models by Boyko and Martins-Mendes categorize risk subjectively, in contrast to PODUS 2020 which predicts absolute risk.

#### Prognostic accuracy

All 4 risk prediction models have been externally validated. The Beulens et al. SR externally validated the models by Boyko and Martins-Mendes [9]. PODUS 2020 was externally validated by the study team [8]. Prognostic accuracy (calibration and discrimination) in validation studies for these models is described in Table 2.

**Table 2:** Performance characteristics of recommended risk prediction models for diabetic foot ulcer development or amputation

**Prediction model; derivation cohort characteristics, size**	**Validation**	**Validation cohort size (n); characteristics**	**Outcome predicted**	**Prediction horizon**	**Discrimination; C-statistic or AUC (95% CI)**	**Overall Calibration Slope**^a^ **(Observed/Predicted)** **(95% CI)**
Boyko et al. (2006) [15]; 95% type 2 diabetes, *n* = 1285	Internal (development cohort)	1285; Veterans in the U.S., 95% with type 2 diabetes seen in primary care clinics, 98% male	DFU	1 year	0.81 (NR^b^)	NR
			DFU	5 years	0.76 (NR)	NR
	External	7624; Patients in Netherlands with type 2 diabetes seen in primary care clinics; 53% male	DFU	5 years	0.81 (0.75, 0.86)	NR
Martins-Mendes et al., original^c^ (2014) [17]; 98% type 2 diabetes, *n* = 644	Internal (development cohort)	644; Patients in Portugal, 98% with type 2 diabetes seen in diabetes foot clinics, 47% male	DFU	3 years	0.8 (0.76, 0.84)	NR
			Amputation	3 years	0.83 (0.78, 0.89)	NR
	External	7624; Patients in Netherlands with type 2 diabetes seen in primary care clinics; 53% male	DFU^3^	5 years	0.78 (0.73, 0.82)	1.56 (NR)
			Amputation	5 years	0.81 (0.74, 0.88)	1.26 (NR)
Martins-Mendes et al., simplified (2014) [17]; 98% type 2 diabetes, *n* = 644	Internal (development cohort)	644; Patients in Portugal, 98% with type 2 diabetes seen in diabetes foot outpatient clinic, 47% male	DFU	3 years	0.79 (0.76, 0.83)	NR
			Amputation	3 years	0.81 (0.74, 0.87)	NR
	External	7624; Patients in Netherlands; 100% type 2 diabetes seen in primary care clinics; 53% male	DFU	5 years	0.77 (0.72, 0.82)	0.97 (NR)
			Amputation	5 years	0.78 (0.71, 0.84)	1.41(NR)
PODUS (2020); type 1 and 2 diabetes, *n* = 8255	Internal (development cohort)	8255; Patients from 4 cohorts in Europe and U.S. with type 1 or 2 diabetes seen in primary and secondary foot clinics; 53% male	DFU	2 years	NR	NR
	External	3324; Patients in U.K. with type 1 or 2 diabetes; 91% type 2 diabetes seen in primary and secondary foot clinics; 57% male	DFU	2 years	0.83 (0.79–0.87)	1.14 (0.99–1.28)

^a^Prior to recalibration; ^b^Not reported; ^c^The model of Martins-Mendes et al. (original) for predicting DFU used physical impairment as a predictor. Since, this variable was not available in the external validation cohort, validation was conducted with the assumption that none of the participants were physically impaired

#### Discrimination

In external validation studies, discrimination was good to excellent for all 4 models (Boyko, Martins-Mendes (original and simplified), and PODUS 2020) predicting DFU, and the 2 models (Martins-Mendes) predicting amputation [9].

#### Calibration

In external validation studies, calibration plots for the models by Martins-Mendes (for DFU and amputation prediction) and PODUS 2020 (for DFU prediction) showed good agreement between observed and predicted absolute risks in the lower quintiles of predicted risk, but observed risk exceeded predicted risk in the higher quintiles [9].

### Usability characteristics of prediction tools

Table 3 and Appendix Table 3 describe variables included, score calculation, and score interpretation for the 4 recommended risk prediction models. The 4 models include 2 to 7 variables which can be obtained by history or chart review (prior DFU, prior amputation, and diabetes complications), physical exam (neuropathy, peripheral arterial disease [PAD], fungal infection, and physical impairment), diagnostic testing in the clinic (visual acuity), and laboratory tests (microbiology to assess for onychomycosis or tinea pedis, and HbA1c). The models by Boyko and Martins-Mendes (original or simplified) require a calculator to determine risk score, but PODUS 2020 score is a simple addition (not requiring a calculator). The models by Boyko and Martins-Mendes (original or simplified) provide a subjective assessment of DFU or amputation risk, but PODUS 2020 quantifies absolute average DFU risk at 2 years of follow-up.

**Table 3:** Variables included in risk prediction models for diabetic foot ulcer development or amputation

Prognostic models (outcome)	Foot related variables					Non-foot related variables				#Variables
	Neuropathy^a^	PAD^b^	Prior DFU	Prior amputation	Fungal infection^c^	Diabetes complications^d^	Poor vision^e^	Physical impairment	HbA1c	
Boyko et al. (2006) [15] (DFU)	✓		✓	✓	✓		✓		✓	6
Martins-Mendes et al. original model (2014) [17] (DFU)		✓	✓			✓		✓		4
Martins-Mendes et al.; original model (2014) [17] (amputation)		✓	✓			✓				3
Martins-Mendes et al.; simplified model (2014) [17] (DFU) or amputation)			✓			✓				2
Martins-Mendes et al.; simplified model (2014) [17] (amputation)			✓			✓				2
PODUS (2020) (DFU)	✓	✓	✓	✓						4

^a^Neuropathy was present if the patient was insensate to 10-g monofilament

^b^Peripheral arterial disease was present if at least one pedal pulse was not palpable

^c^Evaluation for fungal infection included an assessment for tinea pedis and onychomycosis

^d^Diabetes complications included retinopathy, nephropathy, neuropathy, cerebrovascular, cardiovascular, peripheral arterial disease and metabolic abnormalities (ketoacidosis, hyperosmolar coma or any other coma)

^e^Vision was poor if less than 20/40 on testing

## Discussion

Development of a DFU or amputation has severe consequences for the individual and healthcare system [21]. The identification of persons at a high absolute risk for DFU or amputation over a specified time horizon can aid in targeted monitoring and focused prevention efforts, whilst reducing unnecessary resource expenditure on low-risk persons. In this review of reviews, we found 3 SRs describing the performance characteristics of tools predicting DFU development or amputation [9–11]. The SR by Monteiro-Soares et al., published prior to the development of 3 of the 4 top-performing models, did not identify any well-performing tools [11]. The 2 more recent SRs (2021 and 2020), included studies published in PubMed and EMBASE databases over similar time frames. However, these 2 SRs differed in their search criteria, included study populations, and necessity for tool/model validation and likely due to these differences, the 2 SRs identified different tools and reached different conclusions. Thus, in contrast to the low ROB SR by Beulens et al. which identified risk prediction models with a specified time horizon for prediction [9], the moderate ROB SR by Fernandez-Torres et al. mostly identified risk classification systems without a prediction time horizon [10]. Since tools that do not provide a time horizon for risk prediction are less useful for shared clinical decision making, we excluded these from further consideration.

Based on the results of the SRs, we investigated the performance of 4 models predicting DFU development (Boyko, Martins-Mendes [original and simplified], PODUS 2020), and 2 models predicting amputation (Martins-Mendes [original and simplified]) over a specified time horizon [8, 15, 17]. For the models by Boyko and Martins-Mendes, the time frame for risk prediction varied not only between models, but also for the same model between internal and external validation studies [9]. Additionally, categorization of risk, and thresholds used to define levels of risk varied across models without clear rationale. However, all 4 models had good prognostic accuracy, with PODUS 2020 performing best [8, 9].

Clinical usability is a critical consideration for successful widespread adoption of a risk prediction model. Given the time constraints in primary care clinics, the ideal prediction model includes evaluation of a few readily obtainable variables that permit accurate, reproducible, and understandable risk calculation to clinicians and patients. The 4 models included 2 to 7 variables. The models by Boyko and Martins-Mendes include many variables, or variables that take time or resources to obtain. In addition, these tools also require a calculator for risk calculation. These factors decrease the likelihood of widespread use of the Boyko and Martins-Mendes models. In contrast, PODUS 2020 consists of 3 binary variables (neuropathy [1 point], absence of any pedal pulse [1 point], and prior history of DFU or amputation [2 points]) that can be easily measured in the clinic and the scoring is summative [8]. In the PODUS 2020 developmental cohort, 8.5% of all persons had a prior history of DFU or amputation. The risk of DFU at 2 years with scores of 0, 1, 2, 3, and 4 were 2.4%, 6%, 14%, 29.2%, and 51.1%, respectively, with 5.2% of all persons developing a DFU at 2 years. PODUS 2020 has also been externally validated in an independent cohort, in whom 3.9% of persons developed DFU at 2 years. Based on their findings, PODUS 2020 authors concluded that individuals with a score of ≥ 1 (predicted 2-year rate of DFU ≥ 6%) would benefit from preventative treatment [8]. Based on our review, the PODUS 2020 model has the most favorable prognostic accuracy and feasibility characteristics to predict primary or subsequent DFU development. Furthermore, the absolute quantification of DFU risk provided by PODUS 2020 is more easily understood by clinicians and patients, and potentially more informative for shared clinical decision making than models that do not provide a time horizon or categorize individuals by subjective risk categories.

We identified several limitations in the models. For PODUS 2020, prognostic accuracy depends on the ability of clinicians to accurately use a 10 g monofilament and assess for palpable pedal pulses; skills which may vary based on clinicians’ specialty and experience [18]. All studies assessed one-time tool use to predict DFU development or amputation at specified time horizons. In the absence of studies on how risks for DFU change over time, appropriate rescreening intervals for any model are unknown. Most models did not assess if race or ethnicity predicted DFU development, hence the accuracy of these models in predicting DFU in different populations is unknown. Despite the relative simplicity of PODUS 2020, use of this tool in the primary care clinic setting may be challenging as patients and clinicians have competing health care priorities and there are limited time and resources to address them. Lastly, whether tool deployment will better guide referral, subsequent monitoring, or initiation or continuation of treatment to reduce risk of DFU or amputation is unknown.

A limitation of our review is that our search only includedstudies published in English which may have excluded potentially relevant SRs, however, we did not have geographical or date limitations on the search strategy or study eligibility. A strength of our review was that we were inclusive with search, abstraction, analysis, and critique criteria of SRs and we additionally reviewed primary studies of tool development to qualitatively describe clinical feasibility of prognostically accurate models.

### Future research

All current models predicting DFU development include a history of DFU as a risk factor. However, most individuals seen in primary care do not have a history of DFU and are likely at much lower absolute risk of DFU development or amputation. Future studies should develop and validate models to predict development of first DFU. Prior to widespread use, models should be externally validated in the intended healthcare setting to ensure site-specific prognostic accuracy and feasibility. In theory, risk prediction tools identify high-risk individuals for targeted early preventive interventions. Future research should also focus on determining appropriate triage decisions for level of identified risk, and whether such decisions result in improved health outcomes. Additionally, future research is needed to identify DFU risk in racial and/or ethnic minorities who may be at increased DFU risk or have limited access to healthcare. Lastly, research is needed to determine appropriate rescreening intervals for the risk prediction tools and associated incremental benefits and harms of these strategies.

## Conclusions

Four well-performing models discriminate the risk of developing primary or subsequent DFU or amputation in adults with diabetes who are ulcer-free at baseline. Of these, PODUS 2020 has the most favorable prognostic accuracy and is feasible to use in the primary care clinic setting. The PODUS 2020 score predicts average absolute rate of DFU development at 2-years. The rescreening interval and the type or effectiveness of interventions in response to prediction scores to decrease DFU or amputation are unknown.

## Data Availability

Not applicable.

## Appendix

**Table 4:** Search strategy

MEDLINE	
1	Diabetic Foot/ or Foot Ulcer/
2	(diabetic adj1 (foot or feet or ulcer$1)).mp
3	1 or 2
4	Risk Assessment/
5	(assessment$1 or tool$1 or instrument$1 or (objective clinical measures) or valid* or reliab* or scale$1 or score$1 or predict*).mp
6	(screen$ or predict$ or sensitive$ or specific$ or risk factor$ or assess$).ti, ab
7	4 or 5 or 6
8	Orthotic Devices/
9	3 and 7
10	3 and 8
11	9 or 10
12	(systematic review.ti. or meta-analysis.pt. or meta-analysis.ti. or systematic literature review.ti. or this systematic review.tw. or pooling project.tw. or (systematic review.ti,ab. and review.pt.) or meta synthesis.ti. or meta-analy*.ti. or integrative review.tw. or integrative research review.tw. or rapid review.tw. or umbrella review.tw. or consensus development conference.pt. or practice guideline.pt. or drug class reviews.ti. or cochrane database syst rev.jn. or acp journal club.jn. or health technol assess.jn. or evid rep technol assess summ.jn. or jbi database system rev implement rep.jn. or (clinical guideline and management).tw. or ((evidence based.ti. or evidence-based medicine/ or best practice*.ti. or evidence synthesis.ti,ab.) and (((review.pt. or diseases category/ or behavior.mp.) and behavior mechanisms/) or therapeutics/ or evaluation studies.pt. or validation studies.pt. or guideline.pt. or pmcbook.mp.)) or (((systematic or systematically).tw. or critical.ti,ab. or study selection.tw. or ((predetermined or inclusion) and criteri*).tw. or exclusion criteri*.tw. or main outcome measures.tw. or standard of care.tw. or standards of care.tw.) and ((survey or surveys).ti,ab. or overview*.tw. or review.ti,ab. or reviews.ti,ab. or search*.tw. or handsearch.tw. or analysis.ti. or critique.ti,ab. or appraisal.tw. or (reduction.tw. and (risk/ or risk.tw.) and (death or recurrence).mp.)) and ((literature or articles or publications or publication or bibliography or bibliographies or published).ti,ab. or pooled data.tw. or unpublished.tw. or citation.tw. or citations.tw. or database.ti,ab. or internet.ti,ab. or textbooks.ti,ab. or references.tw. or scales.tw. or papers.tw. or datasets.tw. or trials.ti,ab. or meta-analy*.tw. or (clinical and studies).ti,ab. or treatment outcome/ or treatment outcome.tw. or pmcbook.mp.))) not (letter or newspaper article).pt
13	11 and 12
14	Limit 13 to English language
EMBASE	
1	Diabetic Foot/ or Foot Ulcer/
2	(diabetic adj1 (foot or feet or ulcer$1)).mp
3	1 or 2
4	Risk Assessment/
5	(assessment$1 or tool$1 or instrument$1 or (objective clinical measures) or valid* or reliab* or scale$1 or score$1 or predict*).mp
6	(screen$ or predict$ or sensitive$ or specific$ or risk factor$ or assess$).ti, ab
7	4 or 5 or 6
8	Orthotic Devices/
9	3 and 7
10	3 and 8
11	9 or 10
12	(systematic review.ti. or meta-analysis.pt. or meta-analysis.ti. or systematic literature review.ti. or this systematic review.tw. or pooling project.tw. or (systematic review.ti,ab. and review.pt.) or meta synthesis.ti. or meta-analy*.ti. or integrative review.tw. or integrative research review.tw. or rapid review.tw. or umbrella review.tw. or consensus development conference.pt. or practice guideline.pt. or drug class reviews.ti. or cochrane database syst rev.jn. or acp journal club.jn. or health technol assess.jn. or evid rep technol assess summ.jn. or jbi database system rev implement rep.jn. or (clinical guideline and management).tw. or ((evidence based.ti. or evidence-based medicine/ or best practice*.ti. or evidence synthesis.ti,ab.) and (((review.pt. or diseases category/ or behavior.mp.) and behavior mechanisms/) or therapeutics/ or evaluation studies.pt. or validation studies.pt. or guideline.pt. or pmcbook.mp.)) or (((systematic or systematically).tw. or critical.ti,ab. or study selection.tw. or ((predetermined or inclusion) and criteri*).tw. or exclusion criteri*.tw. or main outcome measures.tw. or standard of care.tw. or standards of care.tw.) and ((survey or surveys).ti,ab. or overview*.tw. or review.ti,ab. or reviews.ti,ab. or search*.tw. or handsearch.tw. or analysis.ti. or critique.ti,ab. or appraisal.tw. or (reduction.tw. and (risk/ or risk.tw.) and (death or recurrence).mp.)) and ((literature or articles or publications or publication or bibliography or bibliographies or published).ti,ab. or pooled data.tw. or unpublished.tw. or citation.tw. or citations.tw. or database.ti,ab. or internet.ti,ab. or textbooks.ti,ab. or references.tw. or scales.tw. or papers.tw. or datasets.tw. or trials.ti,ab. or meta-analy*.tw. or (clinical and studies).ti,ab. or treatment outcome/ or treatment outcome.tw. or pmcbook.mp.))) not (letter or newspaper article).pt
13	11 and 12
14	Limit 13 to English language

**Table 5:** Risk of bias ratings for all eligible systematic reviews

Author, Year	Domain 1 Summary: Concerns regarding specification of study eligibility criteria	Domain 2 Summary: Concerns regarding methods used to identify and/or select studies	Domain 3 Summary: Concerns regarding methods used to collect data and appraise studies	Domain 4 Summary: Concerns regarding the synthesis and findings	Overall risk of bias in the review
Beulens, 2021 [9]	Low	Low	Low	Low	Low
Fernandez-Torres, 2020 [10]	Low	Unclear	Unclear	Unclear	Unclear
Monteiro-Soares, 2011 [11]	Low	Low	Low	Low	Low

**Table 6:** Risk prediction models for Diabetic Foot Ulcer (DFU) development or amputation

Tool	Tool Characteristics				
Boyko et al. (2006) [15]	Variables:	HbA1C, vision poorer than 20/40, history of foot ulcer, history of amputation, monofilament insensitivity, tinea pedis, onychomycosis			
	Model:	A1C × 0.0975 + 0.7101 (neuropathy present) + 0.3888 (poor vision)—0.3206 (tinea pedis present) + 0.4579 (onychomycosis present) + 0.7784 (past history of foot ulcer) + 0.943 (past history of lower limb amputation)			
	Outcome Predicted:	DFU			
	Time Horizon:	1 and 5 years			
	Risk Categories:	Quantified by risk score quartiles as below: Lowest quartile: 0.61–1.47 Second lowest: 1.48–1.99 Second highest: 2.00–2.61 Highest: 2.62–5.07			
Martins-Mendes et al. [original] (2014) [17]	Variables:	Physical impairment, PAD complication history, complications count (retinopathy, nephropathy, neuropathy, cerebrovascular, cardiovascular, peripheral arterial disease and metabolic (ketoacidosis, hyperosmolar coma or other coma)), prior DFU			
	Model:	-3.29 + 0.55 × Physical impairment + 0.93 × PAD complication history presence + 0.27 × number of complications count + 1.51 × Previous DFU			
	Outcome Predicted:	DFU or amputation			
	Time Horizon:	3 years			
	Risk Categories:	unclear			
Martins-Mendes et al. [simplified] (2014) [17]	Variables:	Complications count; includes retinopathy, nephropathy, neuropathy, cerebrovascular, cardiovascular, peripheral arterial disease and metabolic (ketoacidosis, hyperosmolar coma or other coma)			
	Model:	Simplified model for predicting DFU -2.86 + 0.46 × number of complications^*^ count + 1.84 × previous DFU Simplified model for predicting amputation -5.35 + 0.61 × number of complications count + 1.91 × previous DFU			
	Outcome Predicted:	DFU or amputation			
	Time Horizon:	3 years			
	Risk Categories:	unclear			
PODUS 2020 [8]	Variables:	Neuropathy, PAD, history of DFU or lower-extremity amputation			
	Model:	Quantifies risk with total potential scores 0 to 4 using the sum of: Score 1 if insensitive to a 10 g monofilament Score 1 if any pedal pulse is absent (dorsalis pedis and posterior tibial pulses on both feet) Score 2 if history of previous ulcer or amputation			
	Outcome Predicted:	DFU			
	Time Horizon:	2 years			
	Risk Categories:	Score 0—average risk is 2.4% (95% CI 1.4% to 3.9%) at 2 years Score 1—average risk is 6.0% (95% CI 3.5% to 9.5%) at 2 years Score 2—average risk is 14% (95% CI 8.5% to 21%) at 2 years Score 3—average risk is 29% (95% CI 19% to 41%) at 2 years Score 4—average risk is 51% (95% CI 38% to 64%) at 2 years			

**Table 7:** Risk classification systems for Diabetic Foot Ulcer (DFU) development or amputation

Tool	Tool Characteristics	
PODUS 2015 [16]	Variables:	Neuropathy, PAD, history of DFU or lower extremity amputation
	Model:	–
	Outcome Predicted:	DFU
	Time Horizon:	Unclear
	Risk Categories:	Moderate risk: neuropathy or PAD High risk: patient’s history of DFU or amputation
Queensland High Risk Foot Form (QHRFF) tool [18]	Variables:	Foot deformity, neuropathy, PAD, previous ulcer or amputation
	Model:	–
	Outcome Predicted:	DFU
	Time Horizon:	Unclear
	Risk Categories:	Low risk: No neuropathy or PAD At risk: Neuropathy or PAD High risk: foot deformity with neuropathy and/or PAD or previous ulcer or amputation or critical PAD
